# Collectanea: Pathology and Practice

**Published:** 1833-10

**Authors:** 


### COLLECTANEA.

### PATHOLOGY AND PRACTICE.

### CHOLERA CURED BY SULPHATE OF QUININE.

Grafe and Walther's Journal (band xix. heft 2,) contains two important papers, detailing a number of cases of cholera cured by large doses of the sulphate of quinine. The first is by Dr. Bluff, of Aix-la-Chapelle. He observes, that when the Asiatic cholera was approaching the Rhenish provinces, he had become persuaded, by reading a number of books, (especially Walther's preface to the German translation of Searle,) that the cholera was an intermittent fever, of the most aggravated character. However, he treated his first cases with venesection, emetics, &c., but they died; and Dr. B. reflecting that, when several persons, suffering under the same disease, are treated in the same manner, without even temporary benefit, it is probable that the physician has adopted an erroneous plan of treatment, resolved to try the effects of the sulphate of quinine in large doses. We shall content ourselves with giving the first case at length.

Th. P., aged nineteen, a weakly factory girl, as she was returning home after her work, on the evening of the 24th September, 1832, was taken ill in the street, where she vomited her food and a sour fluid. When she got home, she vomited water only, and was attacked with severe pain in the belly. At nine o'clock, Dr. Bluff was called to her. She looked weak, her eyes were sunk and heavy, her countenance pale, the point of the nose cold, the tongue had a yellow coat, and was cold at its extremity; her breath was short, her pulse not to be felt; the hands blueish, cold, and covered with a clammy perspiration; there was pain in the abdomen, but no cramps in the calves, and no wrinkles in the skin. Vomiting alternated with diarrhœa, and the evacuations were choleric. Her consciousness was undisturbed, but there was a state of indifference to every thing going on, which could not be mistaken.—℞. Quinæ Sulph. ʒss.; Aq. Meliss., Aq. Menth. Pip. *aa* ℥iij.; Syrup. Papav. Alb. ℥i. M. She was ordered to take a table-spoonful every quarter of an hour, and to use cold water as a drink.

At twelve o'clock the vomiting and diarrhœa had ceased; the patient was very red in the face, and complained of headach. Cold poultices were applied to the head, and the medicines still continued.

At five in the morning of the 25th the patient felt well, and the medicines and cold applications were omitted. She was ordered to drink weak but hot chicken-broth, in small quantities at a time. On the 26th she got up; and on the second day afterwards she returned to her work.

Dr. Bluff details seventeen cases treated in this manner, but occasionally with larger doses of quinine; the recoveries were thirteen, and the deaths four. The ages of the patients varied from twenty months to sixty-six years.

Dr. Bluff mentions a number of cases from the practice of other physicians, showing the striking analogy between ague and cholera, and observes that, when the latter disease broke out at Burtscheid, close to Aix-la-Chapelle, the four persons first attacked were convalescent from ague.

He gives the following table of cholera cases at Aix-la-Chapelle:

| Age. | No. of Cases. | Cured. | Died. |
|---|---|---|---|
| From 1 to 2 years | 12 | 4 | 8 |
| 2 — 3 — | 6 | 3 | 3 |
| 3 — 5 — | 21 | 7 | 14 |
| 5 — 10 — | 30 | 12 | 18 |
| 10 — 20 — | 28 | 22 | 16 |
| 20 — 30 — | 59 | 35 | 24 |
| 30 — 40 — | 64 | 42 | 22 |
| 40 — 50 — | 60 | 32 | 28 |
| 50 — 60 — | 47 | 22 | 25 |
| 60 — 70 — | 48 | 17 | 31 |
| 70 — 80 — | 31 | 9 | 22 |
| 80 and upwards | 11 | None | 11 |
| Total | 427 | 205 | 222 |
| Males | 216 | 99 | 117 |
| Females | 211 | 106 | 105 |

The other paper is by Dr. Kosser, an army physician, who adopted a similar method of treatment in Poland, and with even greater success. In the town of Wreschen, Dr. Kosser had sixteen cases of cholera, of which fifteen recovered: he administered ten grains of sulphate of quinine every two hours, with half a grain of extract of hyoscyamus; and, gradually growing bolder, he diminished the interval between the doses to a quarter of an hour, and even to ten minutes. He also employed frictions, with spirits of camphor, liq. ammon., or ol. terebinth., together with warm fomentations and tepid baths.

Dr. Kosser afterwards had seven more cholera patients, whom he treated with the sulphate of quinine, and six recovered. In one case a sentinel fell down senseless at his post; he had cramps, and cold extremities, with blue hands and face, but did not vomit. Dr. K. gave him ipecacuanha, in doses of five grains, every half-quarter of an hour; the fifth dose caused copious vomiting; in ten hours consciousness returned, and a few hours afterwards he was able to speak. Violent fever now succeeded, accompanied by rigors, heat, and perspiration. The quinine was administered in large doses, and his health was re-established, says Dr. K., without any critical appearances.

### MISTAKES AS TO THE PRESENTATION IN LABOUR.

Nor is it right to wait too long after the efflux of the waters before proceeding to this examination: for the longer you wait the more the uterus contracts on the fœtus, and presses it against the aperture of the pelvis. It may, in that case, happen that the presenting part will swell to such a degree as to change its form, so to express it, and become undistinguishable. It is thus that even experienced accoucheurs have often taken the breech for the cheeks of the child. We ourselves thought, on one occasion, that we had introduced our forefinger into the ear, and imagined that we recognized one of the temporal regions, when in fact the child presented the lower lumbar vertebræ with a spina bifida, which had broken some time before. Another time we shared the mistake of several of our brethren, among whom was one of the most distinguished accoucheurs of the capital, and we despaired of saving a young woman who was in labour, as it seemed to us that she was about to eject through the vulva the intestines and abdominal viscera, and we thought that we could already perceive their gangrenous smell. It may be easily imagined how great was our surprise when suddenly a violent pain put an end to our mistake and our embarrassment. The woman whom we thought lost was very safely delivered of a child that had been dead several days; it was very deformed, and was bent double backwards: the intestinal canal preceded the trunk, because it had escaped from the abdomen, whose weak and slender parietes were not able to confine it.—*Capuron*, *Cours d'Accouchemens*, sixième edit.

### INHALATION OF CHLORINE GAS.

The inhalation of chlorine gas we have tried rather extensively among the workers in flax, suffering from chronic bronchitis. Sixteen of these men I induced to come every evening, after the day's work, to an apartment, the atmosphere of which we impregnated with chlorine, by pouring muriatic acid on manganese. Here they remained at first for a quarter of an hour, and afterwards for about an hour. One individual declared, the second evening, that he had not slept so soundly for several years as he did the night after inhaling; and, on the fifth evening, all the men declared their breathing freer, and the cough considerably reduced. Those who previously could obtain little unbroken sleep had better nights; and others had regained appetite. The plan, from accidental circumstances, was omitted for three evenings. A recurrence of cough and dyspnœa was the speedy result. They gladly therefore returned to the inhalation of the chlorine, and continued it for several weeks, with the most marked advantage. They have since resolved, on the approach of next winter, to take a room for themselves, adjoining their mill or houses, for the purpose of the regular inhalation of chlorine. Two hatters, labouring under similar diseases, joined the flaxmen, and experienced the same benefit.—*Thackrah on the Effects of Arts, &c. on Health and Longevity.*

### WANT OF PULSE IN THE RIGHT ARM.

During a sitting of the Royal Academy of Medicine, a letter was read from Dr. Poujol, of Montpelier, detailing the case of a woman who has no arterial pulsation in any part of the right arm, either at the wrist, at the bend of the arm, or at the axilla; and yet this arm is as well formed as the left; its temperature and sensibility are the same. The woman says, however, that during the severe cold of winter, she feels a numbness and a coolness in the little finger of that hand, which gradually extends to the other fingers. She attributes the absence of arterial pulsation in her right arm to a violent blow with a stone on the elbow, which she received at the age of ten or twelve; at least, before that period she had a pulse in both arms.—*Archives Génerales de Médecine*, Juillet.

### BELLADONNA AS A PROPHYLACTIC AGAINST SCARLET FEVER.

At Monastir, in 1829, scarlatina raged, both among our troops and the inhabitants of the towns and villages where we were quartered. The grand vizier, who had expended much time and money on the discipline of this his favourite *corps d'armée*, gladly accepted my proposal to try the effects of belladonna. As the troops were generally very young men, and totally unaccustomed to narcotics, the dose I gave was comparatively small: thirty-six grains of the extract of belladonna were mixed up with one pound of the extract of liquorice, and ten grains of this were given morning and evening to each soldier. The success of the experiment far exceeded my most sanguine expectations, for not more than twelve men, out of twelve hundred, sickened after this plan was adopted; of these twelve six died, and it is to be remarked, that the disease continued unabated among the inhabitants where the soldiers were quartered, after it had ceased among the latter, although they lived in the same houses.—*Dr. Oppenheim, transl. in Dublin Journal.*

### THE RADICAL CURE OF HERNIA IN TURKEY.

I had an opportunity of witnessing, at Jenetschär (Larissa), this operation performed by surgeon Michalaki of Sagor. The patient was a robust man, about forty years old, who had the hernia for many years, and was now resolved to get rid of it, on account of the inconvenience it caused when he rode. When the operator had convinced himself that the gut could be easily returned, he tied the patient on a board, forming an inclined plane, so that the patient's feet were much higher than his head.

With one hand he pressed against the neck of the sac, so as to prevent the gut from re-entering it, with the other he made an incision into the tumor, extending from about one inch above Poupart's ligament, or two inches below it. He thus brought to view the proper hernial sac, or as he termed it, the bladder of the rupture. This he pulled forcibly with both hands out as far as possible, tied a strong silken string round the neck of the sac near the ring, and cut away the sac below the ligature. The spermatic chord was evidently included in the ligature, which I remarked to him; but he stoutly denied the possibility of his having committed so unfortunate a mistake.—*Ibid.*

### CHOLERA MORBUS ON BOARD A FRENCH FRIGATE.

At a meeting of the Royal Academy of Medicine, a letter was read from M. Guibert, surgeon of the *Melpomene*, relative to the ravages of the cholera among the crew of that frigate, in consequence of its station before Lisbon, and during its passage from Lisbon to Toulon. The frigate was stationed with impunity in the Tagus for three months, although during that period the cholera was at Lisbon. On the 30th of June the crew was still in perfect health. The disease broke out suddenly, attacking a considerable number, and destroying its victims in a few hours. The sick were immediately disembarked, to be treated on land, and the frigate was got under weigh, and set sail July the 3d. Unfortunately it was followed by this scourge, and on the 5th there were thirty-two new patients on board. On the 7th, however, the symptoms began to abate in severity, and the disease in the rapidity of its progress.

M. Guibert is of opinion that the frigate was attacked by this calamity through the same unknown cause that made it ravage Lisbon. In the first cases there were no premonitory symptoms, and no gradations in the disease, but death ensued in a few hours: the evacuations were abundant and frequent, the face and extremities were cold, there was no perceptible mark of the circulation; the other symptoms were blueness, viscous sweats, aphonia, and the most painful cramps in the limbs, and the muscles of the abdomen, the loins, and thorax, and even in the diaphragm itself. Speedy death, or speedy recovery, were the terminations, except that the patient long suffered from general weakness and dyspepsia; sometimes the disease was converted into a typhoid fever, if the reaction had not been decided, or if stimulants had been given too freely.—*Archives Générales de Médecine*, Juillet.

### REMARKABLE CASE OF ABORTION.

A lady, who was the mother of three children, requested the advice of Dr. Taxil, of Brest, together with another practitioner, to ward off, if possible, a threatened miscarriage, the third within fifteen months. In spite of their endeavours abortion took place; the abortive ovum was entirely excluded, and it was found that the funis passed three times round the child's neck, which was so compressed by it as not to be more than two lines in diameter. It was clear that this malposition of the funis prevented the maternal circulation from passing to the child, thus causing a reflux of blood to the placenta, which was very large in proportion to the child. Dr. Taxil observes, that this twisting of the cord round the neck of the child at so early a period of pregnancy had not hitherto been described. He asks if this twisting was not the cause of the miscarriage. He is surprised too that the body of the child should have been properly developed, though the pressure on the neck must have entirely stopped the fœtal circulation. *Ibid.*

### THE HARDENING SYSTEM.

I am afraid that Dr. Underwood's strongly expressed opinion *of the absolute necessity of inuring very young infants to endure the cold air, as essential to their health,* supported as it is by other popular writers, has been productive of great and extensive mischief.

That *pure* air is essential to the health and growth of children, is too evident a proposition to require proof; and that, from the open air, of temperate quality, all children, even very young infants, derive great advantages, is daily manifested by their general healthy appearance, by the colour of their cheeks and lips, and by the firm feel imparted to their muscles. But a belief seems to obtain, that the more cold the temperature of the open air, so much the more pure and bracing will it prove. Now the contrary is rather the fact. It is the temperate quality, not the coldness of the air, which renders it pure and salubrious. Our coldest winds blow from the proverbially unsalutary north and east; and, during the prevalence of these winds, the most severe pulmonary affections, croups, sore throats, swelled glands, &c. continually occur, not only among children, but among adults likewise, who are much xposed to their influence.

It is rather extraordinary that we should be urged to expose our young infants to "very cold and other inclement weather," for the purpose of hardening them, as it is absurdly called, when we find from experience that the young of the irrational creation are injured by such exposure. The gardener, well knowing that "the tyrannous breathing of the north shakes all our buds from blowing," carefully preserves his young plants from the bleak weather; the good housewife secures her young broods of turkeys and other poultry, and the husbandman his tender calves and lambs, from the cold and piercing winds: it is our children alone that we voluntarily expose to the chilling and inclement sky.

Dr. Underwood, indeed, advises that the children should be properly clothed and attended to: this will moderate the evil, but will not cure it. Warm clothing alone is not sufficient to sustain the animal temperature: combined with exercise, indeed, it admirably answers this purpose, but very young infants are incapable of the necessary exercise, and, though warmly clad, soon suffer under the distressing effects of cold. Nor is this consequence of cold upon passive, quiescent subjects, confined to infants. Those who drive or ride in open carriages are usually clothed with as much care to exclude the cold as the infants who are carried in their nurses' arms, but, notwithstanding, frequently suffer extremely from the degree of cold to which they are exposed; and why should children, who are less able to bear such effects of cold, be inured to that which even strong men and women cannot bear with impunity?

True it is that some very robust infants endure the cold in a very remarkable manner, and these are often quoted as examples of the benefit to be expected from the hardening system; but a wise man will be cautious how he follows that as an example which is mentioned only because it is extraordinary. The rules which are to guide our practice should be drawn from what is usual, not from what is uncommon; yet we are too often led away to imitate what is marvellous, and despise that which is more accordant with nature's laws and precepts. Thus, on the evidence of one strong, vigorous infant, the hardening system is applauded and adopted, and we neglect to inquire what numbers have sunk into the silent grave, in the vain attempt to render them, by exposure to the cold, equally vigorous and robust.—*Merriman's Notes on Underwood.*

### CASE OF TRAUMATIC TETANUS.

A boy, aged fifteen, of a bad constitution, had a large phagedenic ulcer on the left heel, for which it was thought necessary to amputate the leg. The first three days passed over favourably, and union by the first intention took place over a considerable surface; but an increased sensibility of the limb, and some convulsive movements in the stump, caused apprehensions of an attack of traumatic tetanus. On the sixth day the patient was seized with a trismus, and all the muscles of inspiration soon shared in the attack. He was bled five or six times, but in vain; the disease invaded the extensors of the arms, and the patient died the eighth day after the amputation.

*Post-mortem examination.* Beyond the stump, at the head of the peroneus, there was a small quantity of pus, and there were traces of inflammation all around it; the sciatic nerve, where it passes through this point, was so injected, that it was of a deep red colour, and this alteration in its appearance extended towards the hip joint for a distant of eight or ten inches. M. Lepelletier pointed this out as a sign of a neurilematic inflammation, which he believes to have been the origin of the local tetanus. In fact, the spinal marrow presented a similar vascularity of the pia mater, but only in the portion corresponding with the origin of the nerves which experienced the direct influence of the wounds, and which confer sensibility and motion on the muscles which were affected by the tetanus. This case, observes M. Lepelletier, confirms an opinion which he has published, namely, that traumatic tetanus is the effect of a neurilematic inflammation.—*Archives Génerales de Médecine*, Juillet.

### CASES OF EASY PARTURITION IN DEFORMED WOMEN.

A woman, aged forty, came to the hospital of the medical school to seek relief for some chronic affection. This unfortunate creature, besides the strangest distortions of her limbs, and a curvature of her spine, had a depression of the sternum, and a humpback; the clear and ineffaceable marks of a disease which she said she had experienced at the age of eighteen. Professor Antoine Dubois asked her if she was married, to which she answered in the affirmative, adding that she was the mother of six children, whom she had brought into the world without the least difficulty. It is therefore extremely probable that her pelvis had not been injured either in its shape or its proportions. Another woman, not less strangely distorted than the one we have just mentioned, became pregnant twenty years ago, in consequence of intercourse with a grenadier; her deformity was so great that, towards the end of her pregnancy, the hip of one side approached the axilla of the other, from the curvature of the spine, and the slope of the pelvis. Nine months afterwards, however, she was delivered in our amphitheatre of a large living child, to the astonishment of the pupils, and especially of some accoucheurs who had condemned her to the Cæsarean knife. We must observe that this woman did not lose the symmetry of her shape till the age of twelve, when the bones of the pelvis were probably solid enough to resist the inroads of the malady which had curved her spine and deformed her limbs. The same woman was delivered again quite naturally in the month of October, 1813, in the presence of Dr. Barbette, our president, and several midwives who were pupils; but she died of general dropsy two months afterwards; and, on the post-mortem examination, we saw that the pelvis, though not regularly formed, was nevertheless sufficiently wide to allow a fœtus of ordinary size to pass through it.—*Capuron, Cours d'Accouchemens*, sixième edit.

---

